# Implementation Insights from the PEACE Pathway Across UK Eating Disorder Services

**DOI:** 10.3390/nu17091532

**Published:** 2025-04-30

**Authors:** Kate Tchanturia, Dimitri Chubinidze, Fiona Duffy, Emy Nimbley, Zhuo Li, Joanna Holliday

**Affiliations:** 1 Department of Psychological Medicine, Institute of Psychiatry, Psychology and Neuroscience, King’s College London, London SE5 8AF, UK; zhuo.li@kcl.ac.uk; 2 Eating Disorders Unit, South London and Maudsley NHS Foundation Trust, London SE5 8AZ, UK; 3 Department of Psychology, Illia State University, Tbilisi 0162, Georgia; 4 School of Health in Social Science, University of Edinburgh, Edinburgh EH8 9YL, UK; fiona.duffy@ed.ac.uk (F.D.); emy.nimbley@ed.ac.uk (E.N.); 5 NHS Lothian Child and Adolescent Mental Health Service, Edinburgh EH16 4TJ, UK; 6 Oxford Health NHS Foundation Trust, Oxford OX4 4XN, UK; joanna.holliday@oxfordhealth.nhs.uk

**Keywords:** eating disorders, autism, implementation, PEACE pathway, reasonable adjustments

## Abstract

Background/Objectives: Autistic individuals with eating disorders (ED) face socio-emotional, sensory, and communication difficulties that influence engagement and treatment outcomes. We examined how the PEACE Pathway—an autism-informed approach to ED treatment—addresses these challenges through tailored adaptations in clinical care. Methods: A qualitative multiple case studies design was employed, drawing data from clinical documentation, stakeholder feedback, and service evaluations. Results: We identified eight core domains essential for implementation: pathway knowledge, assessment and planning, psychological interventions, sensory management, nutritional care, lived-experience feedback, family/community engagement, and staff training. These domains informed the development of the PEACE Self-Assessment Checklist to support the wider adoption of the pathway. Conclusions: The PEACE Pathway offers a structured approach to adapting ED treatment for autistic individuals. The checklist provides practical guidance for implementing autism-friendly adaptions.

## 1. Introduction

### 1.1. Background: Eating Disorders in Neurodivergent Populations

Eating disorders (ED) in neurodivergent individuals, particularly those with autism (terminology preferred by the autistic community) [1], present unique challenges for both patients and clinicians. Standard treatment models do not take into account the sensory, cognitive, and communication differences that can impact engagement and recovery in this population [2]. In particular, autistic individuals may share overlapping cognitive characteristics such as a preference for sameness, cognitive inflexibility, and similarities in social cognition [3].

Emerging evidence suggests that personalised and neurodivergent-affirming approaches can significantly improve treatment outcomes [4,5,6]. However, current interventions are often not fit for purpose and may even exacerbate distress if they overlook autism-specific needs, such as sensory sensitivities and executive functioning challenges [7]. This highlights the need for structured, autism-informed treatment pathways that are co-designed with those with lived experience and that directly target the mechanisms driving mental health difficulties in autistic people.

One such approach is the Pathway for Eating Disorders and Autism developed from Clinical Experience (PEACE), which was developed by the Maudsley team at South London and Maudsley NHS Foundation Trust (SLaM) and King’s College London (KCL). In this paper, we refer to it as the PEACE-Maudsley to reflect its origins and the development of this pathway [8,9].

Over the past six years, PEACE-Maudsley has introduced targeted adaptations across inpatient, outpatient, and day care services, to address key barriers to treatment for autistic individuals with ED. These modifications were informed by research evidence [10,11,12]. Reported outcomes include improved patient–clinician communication [13], enhanced sensory and emotion regulation strategies [14,15,16,17], positive evaluations from both patients [18] and clinicians [19], and reduced intensive care admissions [6].

### 1.2. The Need for a Structured Implementation Framework

International interest in ED-Autism co-occurrence has grown, highlighting the need for structured implementation frameworks. A bibliometric and network analysis by Mota et al. [4] highlights the UK as a leading research hub in this field, with KCL and SLaM at the forefront of innovation. However, despite increasing recognition of these treatment needs, many clinical settings lack formal guidance on integrating autism-informed adaptations into routine ED care [20].

The PEACE-Maudsley was co-designed with key stakeholders, including autistic individuals with EDs, clinicians, carers and researchers to ensure that multiple perspectives informed its development. This participatory approach aligns with implementation science models—such as the implementation research logic model and strategy selection frameworks—which emphasise stakeholder engagement in adapting evidence-based interventions [21,22]. This participatory model aligns with emerging consensus in the literature, which emphasises that future interventions should be co-produced with autistic people, particularly to mitigate harm and ensure meaningful engagement. These frameworks are especially important considering findings that autistic individuals often perceive ED services as inadequate or even harmful when their neurodivergence is not understood or accommodated [23].

Given the successful implementation of PEACE pathway in various clinical settings, it is important to provide clear guidance on core components, challenges, and best practice for new adopters.

### 1.3. Objectives of This Paper

This paper examines key lessons from the development and implementation of the PEACE Pathway, focusing on clinical adaptations that enhance autism-friendly ED care. Grounded in research and real-world clinical experience, it provides a structured approach to implementation, offering practical insights for teams seeking to adopt or expand these adaptations. By bridging research and clinical practice, this paper contributes to making ED treatment more inclusive and effective for neurodivergent individuals.

## 2. Materials and Methods

### 2.1. Study Design

This study employs a qualitative multiple case study approach [24] to examine the implementation and adaptation of the PEACE Pathway across different clinical settings in the UK. This multiple-case design enables in-depth comparison of pathway adaptations across service contexts. The case study method also allows for detailed analysis of how PEACE was integrated, the challenges faced, and the factors that facilitated its implementation.

Three cases are reported:

- PEACE-Maudsley—The original PEACE pilot at SLaM, focusing on clinical adaptation in a specialist eating disorder (ED) service.
- BOB-PEACE—Buckinghamshire, Oxfordshire and Berkshire West—First adaptation of PEACE for community child and adolescent mental health services (CAMHS) across a broader NHS network (Integrated Care System).
- EDAC—Eating Disorders and Autism Collaborative in Scotland—A research and collaboration initiative integrating autistic individuals’ lived experience to guide best practices [25].

Each case represents PEACE implementation at a different site and provides insights into its scalability, adaptability, and sustainability.

This study did not require ethical approval, as it involved secondary analysis of non-identifiable data collected as part of routine service evaluation and clinical implementation. All data used were either publicly available or derived from anonymised internal service documentation and stakeholder feedback.

### 2.2. Data Collection and Analysis

To examine the implementation and adaptation of the PEACE Pathway across different implementation sites, data were collected from multiple sources to ensure a comprehensive understanding of the facilitators, challenges, and outcomes associated with PEACE integration.

The primary data sources included the following:

1. Documentation, such as clinical reports, service protocols, PEACE huddle meeting notes, and internal reports, which captured implementation processes, service adaptations, and ongoing challenges.
2. Stakeholder feedback, including reflections from clinicians, researchers, autistic individuals, and findings from service evaluations.
3. Research outputs, both published and unpublished, including evaluations of PEACE implementation and conference proceedings.

These sources provided insights into real-world implementation processes, allowing for an in-depth examination of how PEACE was integrated and sustained across the three sites.

### 2.3. Development of the PEACE Self-Assessment Checklist

We developed the PEACE Self-Assessment Checklist using data collected from three implementation sites. This process helped us identify eight key components essential for embedding autism-informed adaptations in ED services. We refined the checklist through a co-design approach that included feedback from clinicians, researchers, and autistic individuals with lived experience. We piloted the tool in six outpatient teams and two inpatient services and gathered clinician feedback at each site.

Expert validation reinforced its clinical relevance. One EDAC clinician stated, “This is definitely a useful tool for having a snapshot of where we are at and seeing at a glance the areas that need to be developed”.

Another added, “For years we’ve had conversations in the team about being more accessible. I think this is a really helpful way of structuring those discussions and focusing us on all of the components rather than just the ones we already know need improvement—it’s the ones not yet on our radar that we really need to address”.

This checklist provides a structured approach for assessing service readiness and guiding future implementation efforts.

## 3. Results

We identified eight core domains as essential for embedding autism-informed adaptations into ED services (see Supplementary Table S1 for detailed site comparisons). PEACE-Maudsley, BOB-PEACE, and EDAC each approached these domains differently, tailoring adaptations to their specialist settings. These domains form the basis of the PEACE Self-Assessment Checklist, a structured tool we developed to evaluate service readiness, identify gaps, and guide improvements. We discuss each domain in more de-tail in the following sections.

### 3.1. Knowledge on PEACE Pathway

Familiarity with the PEACE Pathway played a key role in successful implementation. Across all sites, structured training and awareness-building efforts supported staff understanding of PEACE principles, their clinical relevance, and the resources available. In line with this need, clinicians in all three sites emphasised that access to structured learning materials—such as guides, recorded workshops, and online resources—was critical for knowledge retention and effective clinical application. As one clinician reflected, “We have identified the challenge, we have been given some guidance. But let’s say for someone who starts on the ward, very specific guidance would be good […]. So, coming from general guidance to a more specific treatment plan. I think that would be helpful”.

For example, the PEACE-Maudsley team provided 18 monthly face to face and online trainings, followed with annual PEACE conferences for the past five years; BOB-PEACE delivered a series of 16 monthly webinars across the organisation, which were recorded and subsequently made available to all staff via the organisation’s learning and development platform. Situated within a child and adolescent community setting, BOB-PEACE also aimed to extend relevant learning to other key stakeholders, including school staff and local voluntary organisations. EDAC delivered webinars and introduced podcasts for engagement and training purposes.

### 3.2. Assessment and Treatment Planning

Across the three sites, clinicians identified the integration of autism-specific characteristics—drawn from autistic experiences and standardized measures (e.g., Autism Spectrum Quotient (AQ-10) [26], Social Responsiveness Scale (SRS)-based formulation [27,28]—as a key facilitator of individualised care. PEACE-Maudsley fully embedded communication passports [13] and sensory screening [29] into initial assessments to ensure that treatment was tailored to individual sensory, cognitive, and communication needs. BOB-PEACE adapted these tools for CAMHS populations, while EDAC emphasised co-production in developing assessment tools with autistic individuals.

Clinicians described these tools as critical in settings where formal autism diagnoses were delayed or unavailable. As one Maudsley clinician explained, “Our psychologist would report the results of the screening tools. I think it’s very helpful to have working diagnosis for clinical decisions’’.

At BOB-PEACE, clinicians extended the assessment tools across multi-agency settings to promote earlier identification and broader system awareness. Parents also highlighted the impact of clinician sensitivity to potential neurodivergence, with one parent reflecting, “The most helpful thing the PEACE clinician did was spot my son’s potential autism. No one else, including me, would have identified it”.

At EDAC, the team strengthened autism-informed assessment through a co-production approach, involving autistic individuals in developing the tools. Clinicians there highlighted how screening tools not only improved assessment accuracy but also opened up important conversations early in treatment. As one clinician shared, “The use of autism screeners at assessment has been so helpful to open up conversations, conduct more detailed assessment, and make adaptations quickly rather than waiting for treatment to go wrong”.

Clinicians also reflected on the importance of acknowledging autism-related needs even during acute phases of treatment. As one EDAC participant put it, “I do think just yeah, providing the space even early on in treatment when there is, you know, the priority of life and death issues to still create space, of understanding what’s autism and what’s the eating disorder”.

### 3.3. Psychological Treatments and Individualised Support Plans

All three implementation sites emphasized the need for structured therapy adaptations to align psychological treatments with autistic patients’ cognitive and emotional processing styles [30,31,32]. EDAC extended this approach to include ED family therapy focused interventions, developed in response to stakeholder feedback [33].

Key modifications included adjusting session structure and pace (e.g., using visual aids, reducing cognitive load), enhancing communication clarity (e.g., avoiding figurative language, providing written materials, allowing extended processing time), and offering alternative engagement methods, such as visual or sensory-based techniques in place of purely verbal interventions (For freely available resources and tools, see: www.peacepathway.org (accessed on 28 April 2025)). One EDAC clinician noted, “Recognising that we might need to do kind of multiple sessions of appointments rather than trying to do that multi-layered assessment in one go”.

Clinicians consistently stressed the importance of tailoring communication strategies. As one Maudsley team member explained, “[It’s important] to think about whether they’re, you know, if they’re more visual, and to bear in mind things like, you know, using clear and concrete language and not using sort of very flowery language and not using metaphors”.

PEACE-Maudsley for example introduced a brief autism self-report questionnaire and sensory screening in the beginning of the eating disorder programme (inpatient, outpatient and day care services) [29]. This allowed a timely individualised tailored approach.

BOB-PEACE developed additional modular psychoeducational content to support treatment delivery. As a clinician from the team reflected, “Young people found the additional modules we created helpful in building their understanding of how their autism may intersect with their eating disorder and supporting recovery alongside routine treatment”.

Overall, the findings suggest that structured, ongoing training in autism-informed therapy approaches is critical to sustain these adaptations across different service settings.

### 3.4. Sensory Well-Being Management

Addressing sensory challenges was a key priority across all three implementation sites. At PEACE-Maudsley, sensory needs are screened immediately after admission using a brief, pragmatic assessment [29]. This early identification informed structured sensory modifications—such as dedicated quiet spaces, reduced visual clutter, and the use of sensory toolkits—implemented to improve patient comfort and reduce distress. One clinician described this as a turning point in engagement, “And it was just so important in terms of sort of understanding their sensory needs from the very beginning, because we had so many experiences of people saying that they previously disengaged because of the sensory overload that was going on”. In addition to the initial screening, PEACE-Maudsley developed both in-person and online sensory wellbeing workshops to support ongoing care [15,16,29].

BOB-PEACE adopted a similar approach within CAMHS settings, incorporating practical supports and tailored resources to address sensory needs. As part of this work, “supplementary clinician-supported modules around sensory wellbeing and communication passports” were developed to improve treatment adaptation. One clinician also noted the practical value of sensory supports during sessions, stating, “The sensory boxes PEACE created are so useful in sessions with young people to support engagement”.

At EDAC, a more individualised strategy was used, focusing on feedback gathered through a sensory audit tool of the clinical environment, ensuring that adaptations were patient-led and context-specific.

Future improvements should prioritise embedding sensory screening into routine assessment protocols, supporting the consistent application of these adaptations across different service levels and clinical contexts.

### 3.5. Nutritional Management

Supporting nutritional rehabilitation for autistic individuals with ED required careful attention to sensory-related dietary challenges. Across all three case studies, teams emphasised the importance of flexible, individualised approaches to meal planning. PEACE-Maudsley and BOB-PEACE, adjustments were made to textures, flavours, and food temperatures based on patient preferences, helping to reduce mealtime distress and support nutritional intake. A clinician from the Maudsley team explained how screening tools guided these adaptations, “We’d use the sensory screener to help identify who might benefit from being able to use the PEACE Pathway menu, the alternative menu”.

Key facilitators identified across sites included the following:

- Using sensory screening tools to guide meal adjustments.
- Offering alternative meal structures to reduce mealtime anxiety.
- Providing dietitian training on managing sensory-related food aversions [9,34,35].

At EDAC, there was particular emphasis on the need for more structured training, especially in inpatient or group-based settings, where pragmatic strategies were needed to accommodate sensory needs while maintaining clinical goals [35,36].

### 3.6. Lived Experience Network and Feedback

Clinicians reported that involving autistic individuals in shaping clinical practice and service development was an important part of PEACE implementation. At PEACE-Maudsley, input from individuals with lived experience was included in clinical team discussions, such as huddles and case reviews. One clinician described this as central to the team’s evolving practice, “Hearing directly from autistic individuals transformed how we adapted care—it’s essential to include their voices at every stage”. However, clinicians also noted that more consistent and structured ways of involving autistic individuals would be beneficial [37].

In both BOB-PEACE and EDAC, co-production activities were carried out in direct collaboration with autistic people, including the design of training materials and tools. A clinician involved in EDAC training reflected that, “Autistic individuals with lived experience leading the training programme is so insightful and powerful”.

In BOB-PEACE, young people with lived experience contributed to the development and delivery of all training and webinar sessions. The collaborative approach was viewed positively, as one EDAC collaborator noted, “I love the drive EDAC has to work collaboratively with other autistic individuals with lived experience to deliver better clinical outcomes”.

Despite these examples, participants across sites highlighted ongoing challenges. These included limited time and resources for involvement work, and difficulties integrating lived experience into formal clinical planning or service-level decisions.

### 3.7. Family and Community Engagement

Family and autism community involvement in treatment planning and decision-making was a critical component of long-term treatment success. Across all three cases, family engagement was emphasised, although the form and structure of this involvement varied.

At Maudsley, clinicians highlighted the value of offering structured guidance to families, helping them to feel actively included in care processes [38,39]. As one clinician noted, “Family involvement made a significant difference in sustaining treatment gains, but structured guidance was needed for families to feel included”. The PEACE-Maudsley Pathway introduced online family support during COVID in the form of coffee groups and workshops, led by a family therapist with lived experience [40].

These insights align with findings from Bentz et al. (2022) [41], who reported that autistic young people and restrictive-type ED were significantly more likely to require intensified care (such as inpatient or day programmes) compared to their non-autistic peers. Despite this, outcomes like weight restoration and treatment completion were similar across groups, suggesting that—with appropriate adaptations—family-based treatment (FBT) can be effective. However, their findings also emphasise the need to tailor FBT to support families and young people in both their ED and autism-related needs [33,41].

In BOB-PEACE, family therapy was the central treatment model, given the adolescent focus of the service. Families were seen as integral to care delivery, particularly in adapting interventions to reflect the individual needs and preferences of autistic young people. One EDAC clinician reflected, “They’ve found ways to parent their child who’s autistic, and actually we will be really wanting to draw on those strengths and that knowledge from that family […] to think about how we get eating disorder recovery in the context of having an autistic child”.

Family involvement was also central to developing individualised support plans. A parent from the BOB-PEACE site shared, “The PEACE team have brought a unique un-der-standing of my daughter’s autism and anorexia, and the complex relationship be-tween the two. The involvement of the team has been key to a successful outcome”.

Beyond family engagement, all three sites noted the importance of connections with the broader autism community. Partnerships with local organisations helped support peer-led initiatives, such as carer support groups. Both Maudsley and BOB-PEACE developed PEACE-informed parent support groups to address gaps in accessible resources for carers.

In EDAC, community voices also played a role in shaping priorities for support. As one collaborator observed, “It was interesting to hear that there appear to be several autistic voices suggesting peer support. Again, can only applaud having such initiatives as EDAC to bring this to the fore”.

Participants noted that future developments could include the expansion of autistic-led peer support programmes for individuals with ED, alongside efforts to ensure that neurodiversity within the workforce is welcomed, supported, and meaningfully included in service development and delivery.

### 3.8. Staff Training and Development

Clinicians recognised training as central to effective implementation. At PEACE-Maudsley, the team established a training programme that included regular workshops, supervision, huddles, and access to structured learning materials [42]. The Maudsley team also delivers annual conferences on PEACE developments and innovations, which are freely available online [9]. At BOB-PEACE and EDAC, where the pathway has been implemented and further developed, teams highlighted the need to expand training opportunities and emphasised the value of sessions led by autistic individuals with lived experience of ED.

BOB-PEACE developed accessible and sustainable training formats for clinicians, and across all three sites, regular case consultations—often referred to as “huddles”—were identified as essential spaces for clinical reflection and support. These sessions were used to help staff integrate autism-related knowledge into their delivery of ED treatment. One Maudsley clinician noted the impact of these sessions on staff confidence: “I think with the different trainings we had […] it was all so helpful. And I think it really did help staff confidence and knowledge as well”.

Training content focused on practical areas, such as the following:

- Therapy modifications suitable for autistic individuals
- Sensory regulation strategies
- Communication approaches tailored to neurodivergent needs
- The integration of lived experience perspectives

Clinicians consistently valued training that included voices of those with lived experience. At the PEACE-Maudsley conference, one attendee noted, “The lived experience was particularly valuable… so good to see the focus on sensory needs—this is really under-recognised in inpatient services”. An EDAC participant reflected, “Having people with lived experience speak so movingly and openly was incredible”. Similarly, a clinician attending a BOB-PEACE webinar described it as, “The best training I’ve been on that has brought in such richness of insight from neurodivergent young people themselves. This really helped us to try to step into their shoes and consider what adjustments we might need to be making to our treatments and our clinical environments”.

The Maudsley team implemented a comprehensive programme including autism diagnostic training (ADOS-2 and ADI-R), therapeutic modality adaptations (e.g., CBT, DBT, CRT, and CREST), sensory integration strategies, and regular team huddles to embed learning. Feedback from over 100 Maudsley clinicians showed increased confidence in working with this cooccurrence—rising from 46/100 to 68/100—and 97% recommended the training to others [8]. In the BOB-PEACE initiative, 77% of clinicians (*n* = 114) reported observing clinical improvements and/or a reduction in risk following PEACE consultations, while 96% (*n* = 46) reported a better understanding of the needs of the young person they were supporting.

Attendee feedback from PEACE conferences highlighted the pathway’s relevance, describing it as “very timely” and “powerful for those with autism struggling with an ED”. Others reflected on its practical value, noting they were “still thinking how to use this learning to improve patient experience on the ward”.

### 3.9. PEACE Self-Assessment Checklist: A Structured Tool for Implementation

To support scalability and sustainability, we developed the PEACE Self-Assessment Checklist as a structured tool to evaluate service readiness and identify areas for improvement. Drawing on findings from the three case studies, the checklist encompasses eight key domains that capture the essential components of autism-informed ED services.

By offering a practical framework for self-evaluation and targeted adaptation, the checklist is designed to assist services seeking to implement or strengthen PEACE-aligned practices. The final version is presented in Table 1, outlining domains, definitions, assessment criteria, and indicators of implementation.

## 4. Discussion

### 4.1. Key Considerations for PEACE Adoption

Findings from the three case studies demonstrate that structured training, integration of lived experience, and consistent use of tools, such as sensory assessments and communication passports, are central to successful PEACE implementation. Services also benefit-ted from regular staff reflection, environmental adaptations, and support for individual-ised care planning. Building on these insights, we pose two key questions to guide future implementation: (1) What essential elements should be prioritised, and how can they be tailored to diverse treatment structures and patient needs? (2) What resources, training, and structural modifications are necessary to ensure successful implementation [8,9,34]?

The PEACE Pathway, originally developed at SLaM in 2019, was co-designed with input from patients, clinicians, carers, and researchers. The name “PEACE” was selected by patients themselves, reflecting the pathway’s emphasis on personalised, autism-informed care [8,34]. Over time, PEACE has expanded across the UK, with BOB-PEACE (adapted for CAMHS settings) and EDAC (a research-led initiative prioritising lived experience integration) serving as key early adopters. Each case study provided valuable insights into overcoming common implementation challenges, demonstrating both the adaptability and sustainability of PEACE interventions across various clinical environments. Although PEACE has been implemented systematically in the UK, similar autism-informed pathways have not yet been widely reported internationally. A recent bibliometric analysis by Mota et al. (2024) shows that most research in this area originates from UK institutions, such as King’s College London and SLaM [4]. This highlights the need for further international adaptation and evaluation to ensure broader access to autism-informed ED care.

### 4.2. Core Components of PEACE Implementation

Findings from the studies highlight the following core components essential for implementation:

- Familiarity with PEACE pathway knowledge [8,25,34]
- Autism assessment and treatment planning [43]
- Psychological treatment adaptations, including communication passports andsocially assistive tools [13,14]
- Sensory wellbeing management [15,16,17]
- Nutritional management with consideration for sensory needs [35,36]
- Collaboration with individuals with lived experience [25]
- Family and community engagement [40]
- Regular staff training [42]

Multidisciplinary team huddles: Regular, structured meetings enable clinicians to discuss complex cases, refine screening tools, and incorporate lived experience perspectives. These meetings provide opportunities for continuous learning, support, and supervision, enhancing team-wide confidence in adapting treatment to autistic individuals’ needs [18,34].

Communication passports: This tool supports collaborative care by documenting patient preferences, sensory sensitivities, and preferred communication styles. Clinicians reported that using communication passports significantly improved therapeutic alliance and treatment engagement [13].

Sensory regulation strategies: Integrating sensory screening tools early in treatment (e.g., the pragmatic sensory screening tool [29]) facilitates personalized treatment planning through collaborative formulation, addressing both nutritional rehabilitation and emotional regulation challenges [14,15,16,44].

Environmental adaptations: Modifying treatment environments (e.g., reducing noise, minimising visual clutter, access to sensory items, and adjusting lighting) significantly enhances comfort and engagement for autistic patients, particularly in inpatient settings [17].

One of the primary challenges identified across the case studies was clinician confidence in treating EDs within neurodivergent populations. Training programmes—including lived experience-led talks, workshops, public engagement events, research showcases, conferences and webinars—have been instrumental in building awareness and demystifying autism for ED clinicians. Additionally, peer support networks, structured supervision sessions, and access to evidence-based resources have facilitated the integration of neurodivergent-friendly adaptations into clinical workflows.

When implementing PEACE in various service settings, team structures and resource availability may differ, making it essential to tailor resources to each setting to ensure the sustainability of PEACE adaptations. This is consistent with findings from other intervention studies, where adaptations to interventions often occur in response to contextual changes and evolve iteratively [45]. To reduce the risk of compromising intervention effectiveness due to adaptations that may threaten fidelity [46], the PEACE Self-Assessment Checklist—presented in Table 1—offers a structured framework for services aiming to align with PEACE principles and identify gaps in autism-informed practices. This checklist can be a practical tool for self-evaluation and ongoing service improvement, supporting the scalability and sustainability of PEACE adaptations.

### 4.3. Clinical Implications

The findings of this study highlight the complexity of implementing autism-informed adaptations within ED services and the importance of a structured, context-sensitive approach. The PEACE Self-Assessment Checklist, developed through analysis of three implementation sites across the UK, provides a practical framework to support services aiming to align their care with autism-informed principles. By offering a means to evaluate existing practices and identify areas for development, the checklist may assist teams in systematically planning and embedding adaptations that address autistic needs. This tool can be particularly useful for services initiating or scaling up autism-friendly adaptations within diverse clinical settings.

## 5. Conclusions

The PEACE Pathway provides a structured approach to implementing autism-informed adaptations in ED services. Across UK-based sites, it supported patient engagement, individualised care, and clinician confidence through practical adjustments in assessment, treatment, and service delivery.

While the outcomes are promising, challenges, such as resource limitations and variability in local implementation, highlight the need for context-sensitive planning. The findings offer insights relevant to other services considering similar adaptations.

Implications for practice include integrating the PEACE Self-Assessment Checklist into service review processes, enhancing staff training on autism-informed care, and embedding co-production with autistic individuals in service development.

Future work should explore mechanisms to support broader implementation through shared learning networks and policy alignment.

## Figures and Tables

**Table 1:** PEACE Self-Assessment Checklist: a structured tool for evaluating autism-friendly adaptations in eating disorder services.

Category	Definition	Assessment Criteria	Indicators of Implementation
1. Knowledge on PEACE Pathway	Familiarity with the PEACE Pathway, including access to resources and understanding of its principles ➢ PEACE-Maudsley www.peacepathway.org (accessed on 14 April 2025) ➢ PEACE—BOB www.peacepathwaybob.org. (accessed on 14 April 2025) ➢ EDAC—www.edacresearch.co.uk (accessed on 14 April 2025)	☐ Staff have access to PEACE Pathway resources (e.g., conference recordings, book, guides, PEACEBOB showcase). ☐ Staff demonstrate understanding of PEACE Pathway principles.	Staff can articulate key principles of the PEACE Pathway. Evidence of resource utilization in treatment plans or staff discussions. Evidence of collaboration or engagement with wider teams, including neurodevelopmental services.
2. Assessment and Treatment Planning	Focuses on the use of autism-specific tools for assessment and individualized treatment planning	☐ Short assessment screens (e.g., AQ-10, SRS-based formulation) are used. Autism diagnostic pathways are also in place if further evaluation is required. ☐ Reasonable adjustment checklists are used/considered. ☐ Sensory sensitivity screens are consistently documented. ☐ Patients are routinely offered the opportunity to complete a communication passport Individual treatment plans include sensory and communication needs.	Regular use of tools for screening and planning. Comprehensive, individualized treatment plans addressing autism-specific needs. Evidence of reasonable adjustments being made in care plans/treatment delivery
3. Psychological Treatments and Individualized Support Plans	Evaluates adaptations in psychological therapy to align with autistic needs	☐ Therapy formulations are adapted to autistic needs. ☐ Communication strategies in therapy are individualized. ☐ Therapy sessions follow a clear, structured approach and are adapted to autistic needs where possible (e.g., shorter or more frequent sessions, visual guides, etc.) ☐ Consider supplementary sessions focusing on increased understanding of the overlap between autism and EDs and related needs	Documented evidence of therapy adaptations. Positive feedback from autistic individuals on therapy structure and communication.
4. Sensory Wellbeing Management	Addresses sensory-friendly environments and access to sensory tools	☐ Quiet rooms or sensory-friendly spaces are available (Consideration should be given to the overall clinical environment, with an emphasis on reducing sensory stressors (e.g., lighting, noise, smell, cluttered wall space, arrangement of furniture). Environmental modifications might also include tools to enhance communication about the environment (e.g., videos of services, pictures of waiting rooms, or other visual aids to familiarise patients with the space before their visit)). ☐ Sensory tools (e.g., sensory boxes, weighted pads (The use of weighted pads should consider the potential risks for patients with anorexia nervosa), noise-cancelling headphones) are accessible. ☐ Sensory well-being workshops and/or psychoeducation leaflets are offered to patients. ☐ Using sensory needs assessments (i.e., communication passport)	Presence of sensory-friendly spaces and tools. Scheduled sensory well-being workshops as part of treatment plans. Patients’ awareness of their sensory needs and free resources/handouts. Sensory needs are assessed and documented within communication passport and care plans Staff are making conscious effort to reduce sensory stressors like the use of key covers, dimming lights, etc.
5. Nutritional Management	Focuses on accommodating dietary sensitivities and nutritional needs.	☐ Meal plans consider dietary sensitivities and sensory preferences. ☐ PEACE menu guidelines are followed. ☐ Patients report satisfaction with nutritional planning.	Documented dietary adjustments for sensory sensitivities. Positive patient feedback on meal plans.
6. Lived Experience network and Feedback	Ensures incorporation of insights from autistic individuals.	☐ Patient feedback forms or interviews are regularly conducted. ☐ Feedback from autistic individuals informs pathway improvements.	Clear process for collecting and using patient feedback, ensuring people have meaningful opportunities to participate in the design and evaluation of the service. Organisations should also demonstrate how this feedback has influenced and resulted in tangible changes to improve service delivery. Examples of pathway adaptations based on lived experience insights.
7. Family and Community Engagement	Measures involvement of families and community groups in support processes that are autism informed	☐ Support network involved in treatment planning and reviews ☐ Autism community groups collaborate with the organisation and/or are clearly signposted. ☐ Autism specific family support programmes or resources are available.	Regular family involvement in treatment reviews where appropriate. Partnerships with local autism-support organisations.
8. Staff Training and Development	Assesses staff training on autism-specific needs and the PEACE framework.	☐ PEACE resources or materials included routinely in induction of new staff across relevant pathways (ED and neurodevelopmental teams) ☐ Staff participate in PEACE huddles or autism-specific training sessions. ☐ Training materials are readily available. ☐ Staff receive ongoing professional development on autism-friendly practices. ☐ The service adopts an autism-affirming stance, focusing on incorporating autistic strengths in treatment planning and avoiding deficit-based models of autism.	Majority of staff trained in autism-friendly practices. Evidence of ongoing professional development (e.g., workshops, seminars). PEACE website resources are accessed. Training materials available for autism-friendly and autism-affirming approaches. Evidence of autism-affirming practices, including resources celebrating neurodiversity (e.g., pamphlets, online materials), policies reflecting an autism-affirming stance, and feedback demonstrating awareness of neurodiversity-affirming approaches.

## Data Availability

The data supporting the findings of this study consist of internal service documents, clinical reports, and stakeholder feedback not publicly available due to confidentiality and institutional policies. Some resources, such as the PEACE Pathway guidance documents and training materials, are publicly available at www.peacepathway.org (accessed on 28 April 2025).

## Supplementary Materials

The following supporting information can be downloaded at: https://www.mdpi.com/article/10.3390/nu17091532/s1, Table S1: Summary of PEACE implementation and adaptations at PEACE-Maudsley, BOB-PEACE and EDAC.
