# Supplementary material for: Implementation Insights from the PEACE Pathway Across UK Eating Disorder Services
Source: Nutrients. 2025 Apr 30;17(9):1532. doi: 10.3390/nu17091532 (PMC12073639; doi:10.3390/nu17091532)
Supplement: Supplementary file 1 [file nutrients-17-01532-s001.zip › nutrients-3604212-supplementary.pdf]

**Table S1. Summary of PEACE implementation and adaptations at PEACE-Maudsley, BOB-PEACE and EDAC.**

| | PEACE-Maudsley (Specialist adult ED service) | BOB-PEACE (Community child and adolescent ED service) | EDAC-PEACE (Research initiative focusing on lived experience collaboration) |
|---|---|---|---|
| Domain 1: Knowledge on PEACE Pathway | In-person and online training sessions; annual conferences. | Monthly webinars for all staff; outreach to schools and voluntary organisations. | Webinars and podcasts for training. |
| Domain 2: Assessment and treatment planning | Initial assessment by autism screening tool, communication passport, and sensory screening. | Similar tools adapted for CAMHS populations and multi-agency settings. | Co-developing assessment tools with autistic individuals. |
| Domain 3: Psychological treatments and individualised support plans | Tailored session structure and communication strategies based on needs screening. | Additional psychoeducational content to support treatment delivery and emphasis on family involvement. | Systematic adaptations of evidence-based ED interventions. |
| Domain 4: Sensory wellbeing management | Structured environmental sensory support; sensory wellbeing workshops. | Similar sensory support in sessions with young people; supplementary clinician-supported modules. | Patient-led adaptations based on sensory audit of the clinical environment. |
| Domain 5: Nutritional management | Adjustments to textures, flavours, and food temperatures guided by sensory needs. | Adjustments to textures, flavours, and food temperatures guided by sensory needs. | In progress. |
| Domain 6: Lived experience network and feedback | Patient input informs clinical team decisions. | Co-production activities in collaboration with autistic people. | Co-production activities in collaboration with autistic people. |
| Domain 7: Family and community engagement | Structured guidance for family involvement in treatment; family workshops; partnerships with autism community and organisations. | Family therapy as the central treatment model; partnership with autism community and organisations. | Priorities for support shaped by community voices. |
| Domain 8: Staff training and development | Regular training through workshops, supervision, clinician huddles and structured learning materials. | Regular training webinars, clinician huddles, training led by autistic individuals with lived experience of eating disorders. | Training led by autistic individuals with lived experience of eating disorders. |
